# Does the Number of Levels of Decompression Have an Impact on the Clinical Outcomes of Patients With Lumbar Degenerative Spondylolisthesis: A Retrospective Study in Single-Level Fused Patients

**DOI:** 10.7759/cureus.27804

**Published:** 2022-08-09

**Authors:** Glenn A Gonzalez, Daniel Franco, Guilherme Porto, Christopher Elia, Ellina Hattar, Kevin Hines, Aria Mahtabfar, Matthew O'Leary, Lucas Philipp, Elias Atallah, Thiago S Montenegro, Joshua Heller, Ashwini Sharan, Jack Jallo, James Harrop

**Affiliations:** 1 Neurological Surgery, Thomas Jefferson University Hospital, Philadelphia, USA

**Keywords:** single-level fusion, odi, lumbar fusion, evidence-based medicine, decompression levels, degenerative spondylolisthesis

## Abstract

Introduction

The American Association of Neurological Surgeons (AANS) and the Congress of Neurological Surgeons (CNS) 2014 lumbar fusion guidelines for stenosis with degenerative spondylolisthesis (DS) support surgical decompression and fusion as an effective treatment option for symptomatic stenosis associated with DS. The association between the number of levels decompressed in patients with single-level fusion and clinical outcomes has never been published.

Methods

A retrospective analysis of a single-center, prospectively collected database was performed on 77 patients to compare the effect of the number of decompression levels in patients that received single-level fusion surgery. A total of 77 patients met the criteria. Group one had one level decompressed, group two had two levels decompressed, and group three had three or four levels decompressed. All patients received lumbar fusion surgery at a single spinal level. Outcomes at six months included: Substantial Clinical Benefit (SCB) (ΔODI ≥ 10 points); Minimal Clinically Important Difference (MCID) (ΔODI ≥ 5); no MCID (ΔODI <5 points). Student's t-tests, one-way analysis of variance (ANOVA), and post hoc comparison using unpaired two-tailed student's t-test with Holm-Bonferroni correction were performed. *p* -values were ranked from smallest to largest, and alpha level adjustments were made.

Results

A sub-analysis of each group's clinical outcomes showed that patients with two levels decompressed reached greater clinical outcomes. SCB was obtained by approximately 60% (group one: 12.5% vs. group three: 40%) of the patients. A total of 77.6% (38/49) achieved MCID (group one: 62.5% vs. group three: 55%). Single-level fused patients with two levels of decompression showed an improvement of 48% from baseline ODI, as opposed to group one: 17.85% and group three: 21.1%. Patients belonging to group two showed the lowest rate of no improvement. Baseline ODI scores were similar upon presentation (*p*=0.46), and the difference was found among groups after six months of follow-up (*p*=0.009). Post hoc comparison showed statistical significance in the comparison between group two and group three (*p*=0.009, alpha value: 0.017).

Conclusion

The addition of more than two levels of decompression to single-level fused patients might be associated with poor clinical outcomes and spinal instability.

## Introduction

Lumbar degenerative spondylolisthesis (DS) is one of the most common causes of low back pain and is commonly defined as the displacement of one vertebra over a subjacent vertebra, associated with degenerative changes. The prevalence of DS varies from six to nine percent in the general population with a gender predilection observed in women more than men, 8.1% versus 2.7%, respectively [1].

Several non-surgical modalities have been considered before contemplating any surgical options. The AANS/CNS 2014 lumbar fusion guidelines for stenosis with spondylolisthesis support surgical decompression and fusion as an effective treatment option for symptomatic stenosis associated with DS [2-3]. There is a paucity of evidence showing the effect on clinical outcomes of the number of levels of decompression adjacent to a fused spinal level. Literature has shown the association between adjacent segment disease (ASD) and instability as a consequence of multilevel decompressions or decompression adjacent to a fused level [4-5]. However, the consensus regarding decompression adjacent to a fused segment remains ambiguous. Further, in the setting of the aging general population, the chance of degenerative symptomatic spondylolisthesis with multilevel stenosis is likely to increase in the coming years, and therefore, there is a need to establish the likelihood of good clinical outcomes in a combined surgical approach (fusion with adjacent segment decompression) to a challenging disease process.

There is a need for further research regarding decompression adjacent to a fused spinal segment to clarify surgical decision-making in this biomechanical challenge. This study aims to compare the effect of decompression in patients who underwent a concomitant single-level lumbar fusion to evaluate their change in Oswestry Disability Index (ODI) scores before and six months post-surgery.

## Materials and methods

Study design and setting

A retrospective analysis of a single-center prospectively collected database was performed to identify all patients with lumbar DS undergoing a single-level lumbar fusion with concomitant decompression. 

All cases who underwent elective lumbar fusion surgery for DS from March 2018 until August 2019 were reviewed. All cases were carefully evaluated for compliance with EBM guidelines by a group of neurosurgeons led by a senior spine neurosurgeon. Surgical candidates were chosen by meticulously reviewing the North American Spine Society (NASS) criteria indications and following objective patient-reported outcome measures (PROMs).

Participants, variables, and data measurement

Preoperative and six months postoperative ODI scores were collected. Clinical outcomes were divided into a substantial clinical benefit (SCB) defined as ODI change greater or equal to 10 points (SCB ΔODI ≥ 10 points). SCB thresholds for ODI were defined as a net improvement of 18.8 points, a 36.8% improvement, or a final raw score of 31.3 points [6]. The minimal clinically important difference (MCID) represents the critical point of change from baseline defined as an ODI change greater or equal to 5 points (MCID ΔODI ≥ 5). This cut-off for MCID was chosen based on an anchor-based analysis by Monticone et al. that reported a 4.8 point improvement to be an optimal cut-off for this dichotomous outcome (sensitivity 76% and specificity 63%) [7]. No MCID was defined for patients whose ODI improved but did not reach 5 points (ΔODI 1-4 points), and a group showed no change or worsening ODI. The student's t-test was used to compare the mean ODI scores. All ODI scores are displayed as raw scores (0-50 points) and not as percent disability (0-100).

**Figure 1:**
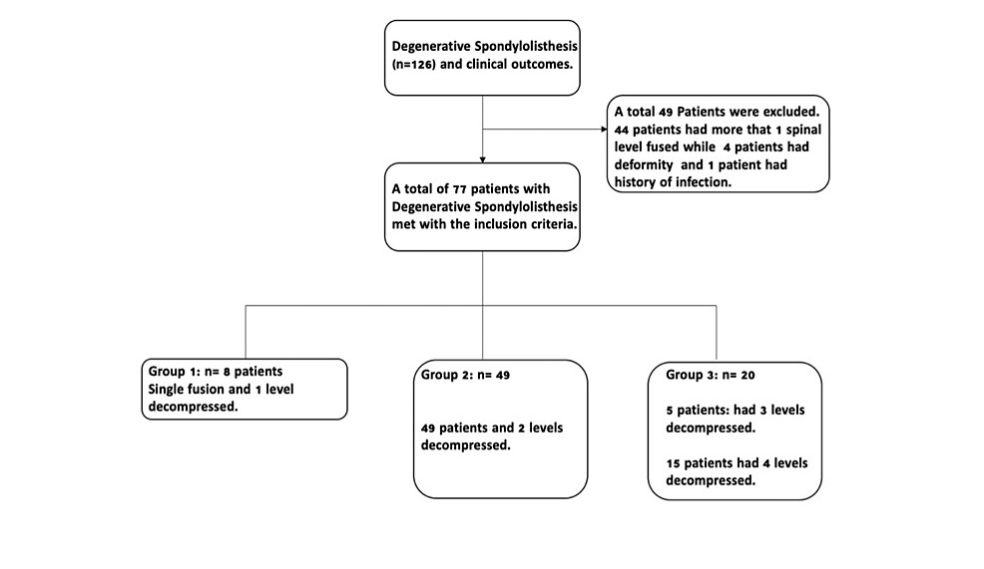
Participants, variables, and data measurement

A total of 126 patients were evaluated. Inclusion criteria were: age ≥ 18 years, the operative treatment was for DS and stenosis, decompression levels were recorded, and patients underwent elective single-level fusion surgery performed by six different spine surgeons. Patients were excluded if they had non-degenerative lumbar spondylolisthesis (e.g., isthmic spondylolisthesis), spinal deformity; if they had trauma, treatment was done non-electively; had more than one level fused, or had previous lumbar surgery. A total of 44 patients were excluded due to more than one level fused. Of the remaining 82 patients, five additional patients (four deformities and one infection) were excluded as well as trauma and non-elective. Patients' symptoms and associated impact on their quality of life must be accurately recorded and documented over time. This is best accomplished using patient-reported outcome measures (PROM). Data points included clinical variables, patient demographics, and PROMS. Seventy-seven (77) DS patients were eligible for inclusion in the final analysis (Figure 1). The single-level fusion occurred at the level of spondylolisthesis associated with instability and/or neurogenic claudication/radiculopathy. The adjacent segment decompression included midline hemilaminectomy with lateral recess decompression/foraminotomy, partial medial facetectomy, or bilateral decompressive laminectomy.

Groups were categorized and stratified by the number of decompression levels. All patients underwent a single-level lumbar fusion with at least one level of decompression. Group one included fused patients with single-level decompression at the fused level. Group two patients underwent two levels of decompression (the fused level and the adjacent level). Group three patients underwent three or four levels of decompression (the fused level and two or three adjacent levels). The decision on the type of decompression (midline-sparring vs. full laminectomy) was at the surgeon's discretion. Preoperative and six-month postoperative ODI scores were compared (Figure 1). Clinical outcomes included: SCB (ΔODI ≥ 10 points), MCID (ΔODI ≥ 5), and no clinical benefit (No MCID) (ΔODI <5 points).

Statistical analysis

Data were collected and transferred for analysis to SPSS 26.0 for Windows (IBM Corp., Armonk, NY), Microsoft Excel 2016 (Microsoft Corporation, Redmond, WA), and Prism 8 (Version 8.4.3 (471); GraphPad Software, Inc, California). The analysis consisted of ODI changes between baseline and six months postoperative. Normality was assessed using visual inspection of variable histograms and Q-Q plots and verified using the Kolmogorov-Smirnov normality test. Preoperative and postoperative mean ODI scores were compared using a paired and unpaired two-tailed student’s t-test; one-way ANOVA was used to compare the mean differences among groups (groups one, two, and three). Post hoc comparison of individual groups was also performed using an unpaired two-tailed student’s t-test with Holm-Bonferroni correction for multiple comparisons. P-values were ranked from smallest to largest, and alpha level adjustments were made to each comparison to decrease the probability of making a type I error (Table 1).

**Table 1:** Comparison of the groups using an unpaired two-tailed student’s t-test with Holm-Bonferroni correction Post hoc comparison of individual groups using an unpaired two-tailed student’s t-test with Holm-Bonferroni correction. ODI: Oswestry Disability Index

	Unpaired two-tailed student’s t-test for delta ODI	Rank	Holm-Bonferroni correction
Group one vs. group two	0.033	2	0.025
Group one vs. group three	0.82	3	0.05
Group two vs. group three	0.009	1	0.017

Descriptive statistics were used to summarize the distribution of the data. All ODI scores were displayed as raw scores (0-50 points) and not as percent disability (0-100). High ODI scores correspond to worse disability and a negative impact on activities of daily living. Statistical evaluations were two-sided, and a p-value < 0.05 was set for statistical significance.

## Results

**Figure 2:**
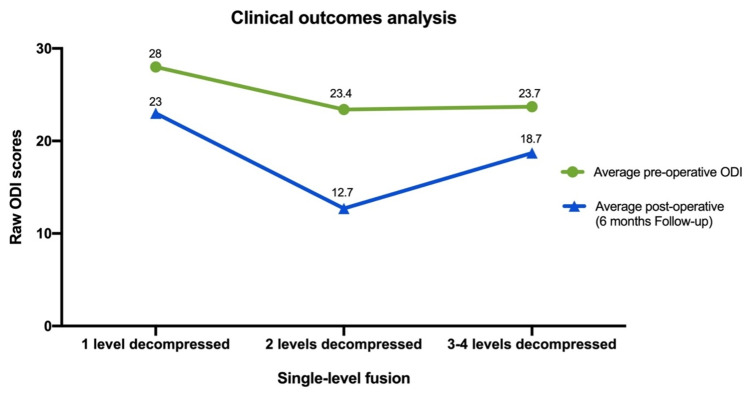
Preoperative and postoperative (six-month follow-up) raw ODI scores on the single-fused population based on the number of levels decompressed ODI: Oswestry Disability Index

A total of eight patients were included in group one, 49 patients in group two, and 20 in group three. There was overall ODI improvement among all our patients in the series. The mean baseline ODI scores in all patients with DS were 24.0 points. At six months, ODI scores averaged 15.3 points among all patients. Baseline pre-surgical ODI scores in patients within group one were 28 points. Baseline pre-surgical ODI scores in patients belonging to group two were 23.6 points, and lastly, group three showed a baseline pre-surgical ODI score of 23.8 points (Table 2, Figure 2).

**Table 2:** Comparison of clinical outcomes among the decompression groups P-values reflect statistical comparisons of variables by groups. One-way ANOVA and a paired two-tailed student’s t-test were used as appropriate. Significance was defined at alpha=0.05. ODI: Oswestry Disability Index, SCB: Substantial Clinical Benefit, MCID: Minimal Clinically Important Difference; ANOVA: Analysis of Variance

	Group one n=8	Group two n=49		Group three n=20	P-values (one-way ANOVA)
Preoperative ODI scores	28 ± 7.7	23.4 ± 8.7	23.7± 9.6		P=0.46
Postoperative ODI	23 ± 9.1	12.7± 8.6	18.7 ± 9.2		P= 0.009
Delta ODI	5.14 points	10.7 points	5 points		P=0.01
SCB	12.5% (1/8)	59.2% (29/49)	40% (8/20)		-
MCID	62.5% (5/8)	77.6% (38/49)	55% (11/20)		-
No MCID	37.5% (3/8)	24.4% (12/49)	45% (9/20)		-
P-values (paired two-tailed student’s t-test)	p=0.10	p<0.001	p=0.008		-

A significant improvement in ODI scores was observed in all patients, preoperative ODI (24.01 +/- 8.83) and postoperative ODI (15.29+/- 9.19) (p<0.0001). No significant improvement was seen in group one: 28 ± 7.7 ODI to 22 ± 9.1 (p=0.10). However, significant improvement was noted in group number two: 23.6 ± 8.7 to ODI 13.1± 8.6. (p<0.001) and in group three: 23.7± 9.6 ODI to 18.7 ± 9.2 (p=0.008) (Table 2, Figure 2).

**Figure 3:**
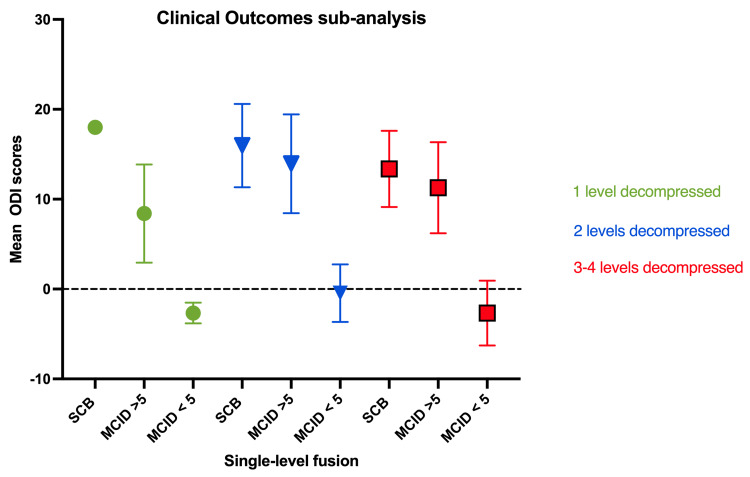
Clinical outcomes sub-analysis on the single-level fused population based on the number of levels decompressed The green circle represents the data of one patient. ODI: Oswestry Disability Index, SCB: Substantial Clinical Benefit, MCID: Minimal Clinically Important Difference

Most patients were female, 66.24% (51/77), with a mean age of 65 ± 8.7 years. A total of 12.5% (1/8) of the one-level decompression (group one) reached SCB ΔODI ≥ 10, while 62.5% (5/8) achieved MCID (ΔODI ≥ 5) and 37.5% (3/8) of patients did not achieve MCID (ΔODI < 5). For group two, 59.2% (29/49) reached SCB, 77.6% (38/49) achieved MCID and 22.4% (11/49) of patients did not achieve MCID (ΔODI < 5). Finally, in group three, patients with three or four levels of decompressed had 40% (8/20) reached (SCB) and 55% (11/20) achieved (MCID). Group three had 45% (9/20) of patients not reach MCID (Table 2, Figure 3).

An overall ODI improvement was seen after six months of follow-up [8]. A thorough analysis of the presurgical versus postsurgical changes in ODI was performed individually over the three studied groups. No statistical significance was obtained in patients who received single-level decompression (p=0.10), as opposed to the level of significance observed in the groups of patients who had two and three or four levels of decompression (p<0.001, p=0.08, respectively).

**Figure 4:**
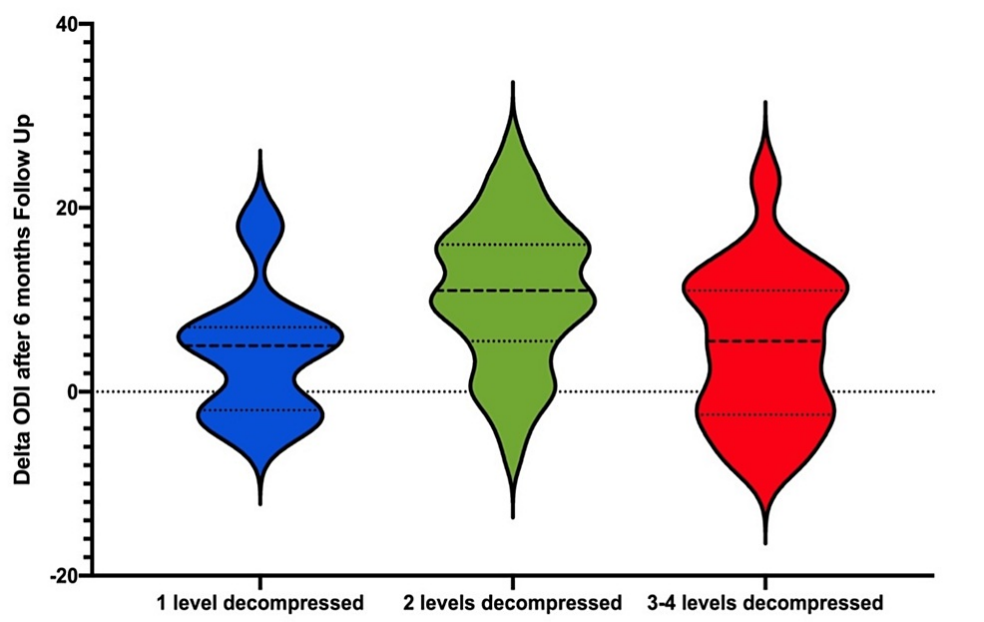
Violin plot for the distribution of change in ODI, stratified by the number of levels of decompression in patients with degenerative spondylolisthesis versus postoperative ODI scores after the six-month follow-up Violin plot upper/lower bounds are the 25% and 75% limits (interquartile range) and are represented by dotted lines. Solid lines represent mean delta ODI. The width of the violin indicates the distribution of the change in the patient's ODI after six months of follow-up. ODI: Oswestry Disability Index

Upon presentation, the mean baseline ODI scores showed no significant difference in mean scores (p=0.46) among the three groups, indicating a similar baseline. A statistically significant difference was found between the three groups' preoperative and postoperative ODI scores at six months (p=0.009). The mean ODI difference among the groups was also statistically significant (p=0.01) (Table 2, Figures 3-4). Post hoc comparison of individual groups using an unpaired two-tailed student’s t-test with Holm-Bonferroni correction showed statistical significance among clinical outcomes of patients with two-level decompression over patients with three to four-level decompression. However, there was no statistical significance in comparing other groups (Table 1).

## Discussion

Lumbar DS is a condition commonly encountered by the spine surgeon, which can be managed surgically and non-surgically. When surgery is considered, the benefits of decompression and fusion have been established in the literature [9-10]. Numerous studies have been performed to compare the effect of decompression and fusion in patients with lumbar spinal stenosis due to DS [11-12]. Despite the efforts to compare the impact of the different types of decompression on patients with DS, there is no consensus in the literature on the impact of decompression multiple levels adjacent to a single-level lumbar fusion for DS [4].

Using an unpaired student’s t-test with Holm-Bonferroni correction, the authors were able to show that better clinical outcomes were achieved in the single-level fused population with two levels decompressed over the population of patients with one or three or more levels of decompression. This does not necessarily insinuate that an adjacent level of decompression portends a more favorable outcome than single or multi-level (three to four levels) decompression; rather, this should be interpreted that an adjacent level of decompression without fusion may provide the patient with significant clinical improvement [13]. Sengupta et al. established that decompression relieves radicular symptoms and neurogenic claudication, whereas fusion relieves mostly back pain by eliminating instability [14-15].

The literature regarding adjacent level decompression in the setting of DS remains conflicted with some advocating multi-level decompression propagating instability and ASD [4-5,16-17]. On the contrary, multi-level fusion has also been shown to potentiate ASD when compared to single-level fusion and leads to significantly higher hospital costs, morbidity, and mortality. For this reason, different treatment goals should be considered in cases where multiple stenosis levels are found [13,18]. In some scenarios, central decompression on top of the fused segment can affect the integrity of the posterior ligamentous complex as well as a partial destruction of the facet complex, leading to potential instability, worsening back and leg pain, and potentiating the need for further surgical intervention [4].

The presurgical and postsurgical ODI scores were obtained and compared for each of the groups. Patients that received two levels of decompression (group two) showed better clinical outcomes than patients with either one (group one) or more than three (group three) levels of decompression (Figures 2-3, Table 2) [11]. Furthermore, a sub-analysis of each group's clinical outcomes was performed. It was observed that patients with two levels of decompression showed greater clinical outcomes after six months of follow-up, and SCB was obtained by approximately 60% (group one: 12.5% vs. group three: 40%) of the patients. Similarly, a total of 77.6% (38/49) achieved MCID (group one: 62.5% vs. group three: 55%). Single-level fused patients with two levels of decompression showed an improvement of 48% from baseline ODI, as opposed to group one: 17.85% and group three: 21.1% [19-21].

In this study, patients received follow-up after six months, and an improvement of 10.7 points in ODI was obtained in the population of patients with two levels of decompression (23.4 ± 8.7 to 12.7± 8.6; p<0.001) (Table 1, Figure 4). Other studies have also suggested that the combination of decompression and fusion led to superior outcomes compared to decompression alone [11]. Among the groups of patients that did not reach MCID after six months, patients belonging to group two showed the lowest rate of no improvement (Table 1, Figure 3). In previous studies, Fraymoyer et al. have established that morbidity associated with laminectomy and fusion increases as a function of age and magnitude of the operation [22]. Our data provides continued, objective evidence to the often-controversial biomechanical principle of adjacent segment decompression to a fusion, within reason, respecting segment stability, the integrity of the facet complex, transitional segments, as well as patient protoplasm. Lastly, although the discussion of degenerative spinal deformity correction is beyond the scope of this paper, it is imperative to respect and take these parameters into consideration during surgical planning.

Limitations

As with all studies, our study is not without limitations. The retrospective nature of the study is in itself a limitation. There is also bias with regards to surgical techniques and type of decompression performed given that the surgeries were performed by six different spine surgeons. Although pelvic parameters and lumbar lordosis play an important role in the development of ASD, we excluded patients with a spinal deformity given that ASD and pelvic incidence - lumbar lordosis (PI-LL) mismatch is beyond the scope of this study. Another limitation is the six-month follow-up time. Ideally, we would like to have at least a two-year follow-up to assess the progression of symptoms or development of instability [23]. In this study, we did not study the association between the number of stenotic levels radiographically detected with the number of decompression levels. The degree of listhesis along with the degree of spinal and lateral recess stenosis should be investigated to further evaluate the effect of decompression levels on clinical outcomes.

To our knowledge, no such prior study has been conducted. Our results reflect findings at a single institution, so external validity was limited. Furthermore, results may have been influenced by the population's small sample size, different decompression techniques, or different surgeons.

## Conclusions

Patients with two levels of decompression showed greater clinical outcomes at the six-month follow-up. SCB was obtained by approximately 60% while MCID was achieved in 77.6% of the patients. Single-level fused patients with two levels of decompression showed an improvement of 48% from the baseline ODI. The addition of more than two levels of decompression to single-level fused patients might be associated with poor clinical outcomes and spinal instability.
